# *Dolphin Morbillivirus *and *Toxoplasma gondii *coinfection in a Mediterranean fin whale (*Balaenoptera physalus*)

**DOI:** 10.1186/1746-6148-8-20

**Published:** 2012-03-07

**Authors:** Sandro Mazzariol, Federica Marcer, Walter Mignone, Laura Serracca, Mariella Goria, Letizia Marsili, Giovanni Di Guardo, Cristina Casalone

**Affiliations:** 1 Department of Comparative Biomedicine and Food Science, University of Padova, AGRIPOLIS - Viale dell'Università, 16, 35020 Legnaro, (PD), Italy; 2 Department of Animal Medicine, Production and Health, University of Padova, viale dell'Università 16, 35020 Legnaro, (PD), Italy; 3 Istituto Zooprofilattico Sperimentale del Piemonte Liguria e Valle d'Aosta, via Bologna 148, 10154 Torino, Italy; 4 Department of Environmental Science, University of Siena "G. Sarfatti", via P.A. Mattioli 4, 53100 Siena, Italy; 5 Department of Comparative Biomedical Sciences, University of Teramo, piazza Aldo Moro 45, 64100 Teramo, Italy

**Keywords:** Dolphin *Morbillivirus*, *Toxoplasma gondii*, Fin whale, DDT, Mediterranean Sea

## Abstract

**Background:**

Although *Morbillivirus *and *Toxoplasma gondii *have emerged as important pathogens for several cetaceans populations over the last 20 years, they have never been identified together in a Mysticete. In particular, morbilliviral infection has been never described in the Mediterranean fin whale population.

**Case presentation:**

On January 2011 an adult male of fin whale (*Balaenoptera physalus*) stranded along the Tyrrhenian coastline of Italy. During necropsy, tissue samples from heart, skeletal muscle, mesenteric lymph nodes, liver, spleen, lung, and kidney were collected and subsequently analyzed for *Morbillivirus *and *Toxoplasma gondii *by microscopic and molecular methods. Following the detailed necropsy carried out on this whale, molecular analysis revealed, for the first time, the simultaneous presence of a *Dolphin Morbillivirus *(DMV) and *T. gondii *infection coexisting with each other, along with high organochlorine pollutant concentrations, with special reference to DDT.

**Conclusion:**

This report, besides confirming the possibility for Mysticetes to be infected with DMV, highlights the risk of toxoplasmosis in sea water for mammals, already immunodepressed by concurrent factors as infections and environmental contaminants.

## Background

Among the several threats to which free-ranging cetaceans are exposed, *Morbillivirus *and *Toxoplasma gondii *are believed to represent a serious hazard to their health and conservation [1]. Nevertheless, morbilliviral infections have been rarely described in mysticetes [2-4], while *T. gondii *has been also reported as a disease-causing protozoan agent in immunocompromised Odontocetes [5,6] affected by a severe meningo-encephalitis, as recently documented in the Mediterranean striped dolphin (*Stenella coeruleoalba*) population [7].

## Case presentation

**Figure 1:**
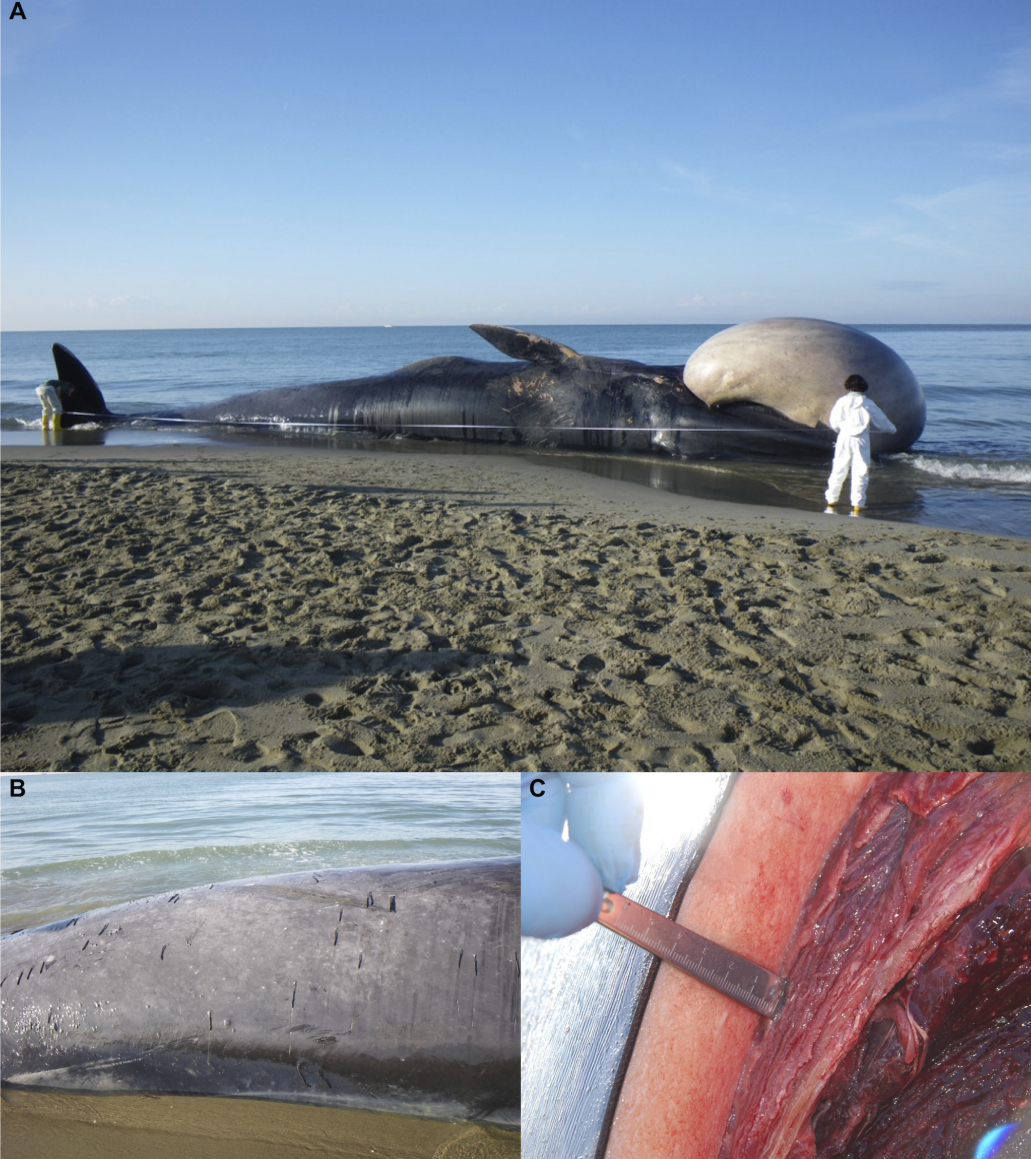
**Steps of the necropsy carried out on the stranded whale**. Biometrical measurements were taken before starting *post-mortem* analyses (A). A severe cutaneous parasitic infestation by *Pennella* spp. (B) was evident during external examination and a poor body condition was assessed due to a reduction of the blubber layer (C).

An adult male fin whale (*Balaenoptera physalus*) was found stranded dead (length: 16.7 m; estimated weight: 25,000 kg) on January 25, 2011 (Figure 1A). Using a photo-identification method, the animal matched with one whale that was observed swimming slowly in shallow waters, in front of the coast of Tuscany (Tyrrhenian Sea), Italy, close to the cost of Follonica (GR) on January 16, 2011, as well as in front of the tourist port of Viareggio (PI) on January 23, 80 km north of the first sighting area. A detailed *post-mortem *examination was performed on site (43°44'42"N,10°16'35"E) within 24 h after stranding, following specific necropsy protocols developed for large whales [8,9]. Sampled tissues were preserved in 10% neutral buffered formalin for histopathology, refrigerated for microbiology and parasitology, and frozen for biomolecular and ecotoxicological investigations. For histopathology, tissues were paraffin-embedded, sectioned at 4 μm, and routinely stained with hematoxylin and eosin (HE). Selected tissue sections from the main organs were also stained by different histochemical techniques (PAS, Prussian blue, Masson's trichrome, Gram). Microbiological, immunohistochemical and biomolecular investigations for the main cetacean pathogens *(Morbillivirus, Herpesvirus, Brucella spp*., and *T. gondii*) were also performed on all major organs. Specifically, we utilized RT-PCR techniques targeting a highly conserved *Morbillivirus *nucleoprotein gene sequence (amplicon size 287 bp) and PCR techniques amplifying a conserved region of the nss-rRNA gene (approximately 300 bp in size) of coccidian parasites (Apicomplexa). Furthermore, immunohistochemical labeling for *Morbillivirus *and *T. gondii *antigens was attempted on molecularly positive tissues [7].

**Figure 2:**
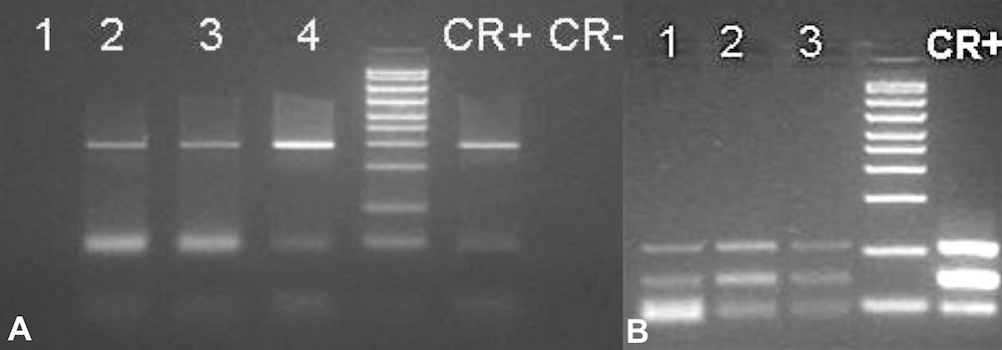
**Molecular analyses yielded a positive result for *Morbillivirus*-specific genome sequences**. RT-PCR targeting *Morbillivirus *nucleoprotein (N) gene gave positive results from the whale's spleen (lane 2), liver (lane 3), and lung (lane 4), while the heart was negative (lane 1); positive and negative controls (C + and C-, respectively) are also shown in figure A along with the DNA ladder marker. Each result was confirmed by means of both sequence and RFLP: the latter is shown in figure B with the positive reaction for spleen (lane 1), liver (lane 2), and lung (lane 3). Immunohistochemistry (IHC) using a commercially available mouse monoclonal antibody (MoAb) solution (1:500) against canine distemper virus (CDV) nucleoprotein antigen (VMRD Inc), which recognizes the same epitope from different members of the *Morbillivirus *genus (including DMV) was negative, possibly due to bad tissue preservation.

**Figure 3:**
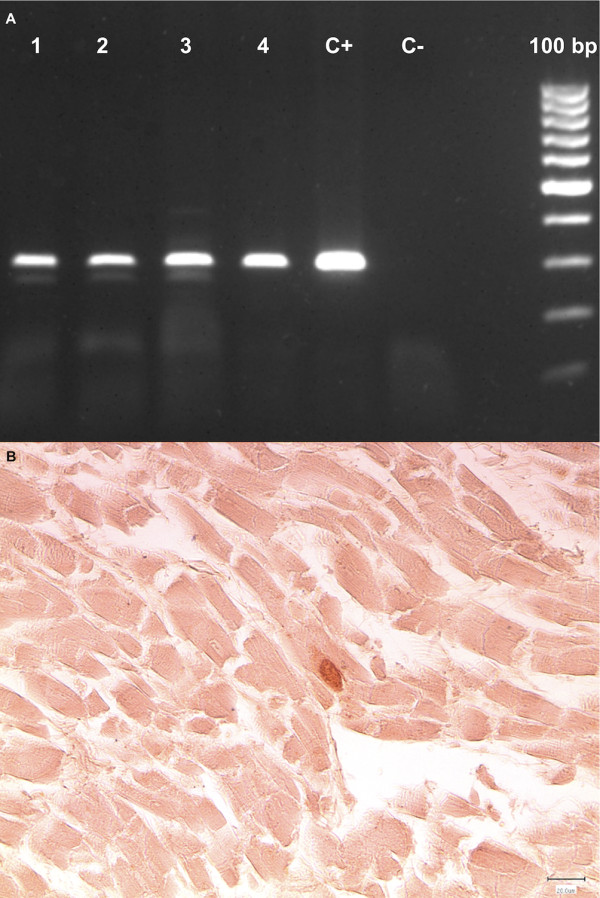
**Molecular and immunohistochemical studies confirmed the presence of *T. gondii *in the whale's tissues**. PCR techniques amplifying a conserved region of coccidian parasites yielded positive results for *T. gondii *in the heart (lane 1), skeletal muscle (lane 2), mesenteric lymph node (lane 3), and kidney (lane 4); positive and negative controls (C + and C-, respectively) are also shown in figure A along with the DNA ladder marker. IHC by means of a commercially available goat polyclonal anti-*T. gondii *antiserum solution (1:1000; VMRD Inc, Pullman, WA) showed rare *T. gondii *cysts embedded into both the myocardial (B; 400×, bar = 20 μm) and the renal tissue.

The whale showed quite an advanced *post-mortem *decomposition (code 3) and a compromised nutritional status (Figure 1A), as documented by the reduced mean thickness (4.2 cm) of the blubber layer (Figure 1C) and by the absence of food in the gastric chambers. A severe parasitic infestation by Pennella spp. diffusely involved the animal's skin (Figure 1B). Opening of major body cavities showed a mild bilateral hydronephrosis, secondary to partial obstruction of ureters and pelvis by calcified parasitic remains (presumably, *Crassicauda *spp.), which were macroscopically assessed. Other relevant pathological findings included massive hepatic and splenic enlargement, congestive mesenteric lymphadenopathy, and mild diffuse fibrinous peritonitis. Microscopic examination of the organs also showed erythrophagocytosis in the spleen. Biomolecular investigations yielded positive results for DMV-specific genome sequences from the whale's lung, spleen, and liver (GenBank accession nr: EF469546.1), with *T. gondii *genomic sequences being simultaneously detected in renal, cardiac and musculoskeletal tissues, as well as in mesenteric lymph nodes (GenBank accession nr: AY663792) (Figures 2 and 3). Specificity confirmation of each result was assessed by means of both sequence and RFLP analysis for DMV and sequence analysis for *T. gondii*, respectively (Figure 2). Rare and isolated *T. gondii *cysts could be also immunohistochemically labeled in kidney and heart (Figure 3), with immunohistochemical staining for morbilliviral antigen being negative in all examined tissues. Finally, ecotoxicological investigations detected high organochlorine (OC) pollutant levels in blubber (total PCBs: 21269.16 ng/g lipidic weight; total DDTs: 23720.94 ng/g lwt).

The present study represents the third report of *Morbillivirus *infection in a fin whale worldwide [2], thus confirming the susceptibility of Mysticetes to this virus, as also suggested by the seropositivity previously described in an Icelandic fin whales [3] and in a minke whale from the Mediterranean Sea [4]. Furthermore, this report is a further proof of the diffusion of morbilliviral infection among cetaceans of the Mediterranean Sea basin, namely pilot whales (*Globicephala melas*), striped dolphins, and bottlenose dolphins (*Tursiops truncatus*) along Spanish, French and Italian coastlines [10-13]. During the dramatic morbilliviral epidemic in the Mediterranean Sea from 1990 to 1992, coinfection with *T. gondii*, an opportunistic pathogen for cetaceans, was reported in striped dolphins [14]. In Mysticetes, the only previous report of infection is limited to seropositivity against *T. gondii *in a humpback whale (*Megaptera novaeangliae*) from the Atlantic Ocean [15]. To the best of our knowledge, this is the first DMV and *T. gondii *coinfection described in a baleen whale. In our fin whale specimen, the molecular identification of *T. gondii *supports the hypothesis of a severe impairment of the immune system, likely induced by the coexisting morbilliviral infection [2]. In contrast to what established during the investigations on the 2006-2008 morbilliviral epidemic in the Mediterranean Sea [16], the consistent body concentrations of OC contaminants measured in the fin whale under study, which were higher than those found in free-ranging animals [17,18], were considered as a likely worsening factor, accompanied by concurrent kidney dysfunction and prolonged fasting, similarly to what occurred during the 1990-1992 epidemic [2]. Furthermore, the absence of evident pathological changes related to *T. gondii *in the heart and in the mesenteric lymph nodes, which appeared non-compromised, suggests an acute protozoan infection.

The transmission pathways through which cetaceans acquire *T. gondii *infection are currently unknown, although *T. gondii *oocysts have been recently demonstrated in run-off waters, shellfish, and filter-feeding fish [5,19,20]; furthermore, oocysts may survive in sea water and remain infective for up to 6 months [21]. No data are available on the route of transmission of *T. gondii *infection in the fin whale described here; however, the animal may have acquired the pathogen while swimming in shallow waters in the days immediately preceding the stranding, with such possibility highlighting the risk of toxoplasmosis for immunodepressed mammals in the marine environment.

## Conclusion

Considering the features and the size of the Mediterranean fin whales' population [22,23], DMV and *T. gondii*, especially when occurring in association to each other and under the influence of anthropogenic activities on cetacean populations living in Mediterranean waters [24], may pose a serious threat for this already endangered species.

## Competing interests

The authors declare that they have no competing interests.

## Authors' contributions

Necropsy and microscopic examinations were performed by SM along with LM, FM and WM; FM and LS analyzed tissue for parasites; MG, GDG and CC studied viral infections by PCR as well as by immunohistochemistry; LM carried out toxicological examination on sampled tissues and coordinated photo-ID matching; the manuscript was prepared and critically discussed by SM, GDG and CC with the contribution of all the remaining authors. All authors read and approved the final manuscript.
